# Elevated BMI reduces the humoral response to SARS‐CoV‐2 infection

**DOI:** 10.1002/cti2.1476

**Published:** 2023-12-03

**Authors:** Marcus ZW Tong, Julian DJ Sng, Meagan Carney, Lucy Cooper, Samuel Brown, Katie E Lineburg, Keng Yih Chew, Neve Collins, Kirsten Ignacio, Megan Airey, Lucy Burr, Briony A Joyce, Dhilshan Jayasinghe, Christopher LD McMillan, David A Muller, Anurag Adhikari, Linda A Gallo, Emily S Dorey, Helen L Barrett, Stephanie Gras, Corey Smith, Kim Good‐Jacobson, Kirsty R Short

**Affiliations:** ^1^ School of Chemistry and Molecular Biosciences The University of Queensland St Lucia QLD Australia; ^2^ School of Mathematics and Physics The University of Queensland St Lucia QLD Australia; ^3^ Department of Biochemistry and Molecular Biology Monash University Clayton VIC Australia; ^4^ Immunity Program, Biomedicine Discovery Institute Monash University Clayton VIC Australia; ^5^ QIMR Berghofer Centre for Immunotherapy and Vaccine Development and Translational and Human Immunology Laboratory, Infection and Inflammation Program QIMR Berghofer Medical Research Institute Herston QLD Australia; ^6^ Department of Respiratory Medicine Mater Health Brisbane QLD Australia; ^7^ Department of Biochemistry and Chemistry La Trobe Institute for Molecular Science, La Trobe University Bundoora VIC Australia; ^8^ Australian Infectious Diseases Research Centre The University of Queensland St Lucia QLD Australia; ^9^ School of Health University of the Sunshine Coast Petrie QLD Australia; ^10^ Mater Research The University of Queensland South Brisbane QLD Australia; ^11^ University of New South Wales Medicine Kensington NSW Australia; ^12^ Obstetric Medicine Royal Hospital for Women Randwick NSW Australia

**Keywords:** antibody immunity, BMI, obesity, overweight, SARS‐CoV‐2

## Abstract

**Objective:**

Class III obesity (body mass index [BMI] ≥ 40 kg m^−2^) significantly impairs the immune response to SARS‐CoV‐2 vaccination. However, the effect of an elevated BMI (≥ 25 kg m^−2^) on humoral immunity to SARS‐CoV‐2 infection and COVID‐19 vaccination remains unclear.

**Methods:**

We collected blood samples from people who recovered from SARS‐CoV‐2 infection approximately 3 and 13 months of post‐infection (noting that these individuals were not exposed to SARS‐CoV‐2 or vaccinated in the interim). We also collected blood samples from people approximately 5 months of post‐second dose COVID‐19 vaccination (the majority of whom did not have a prior SARS‐CoV‐2 infection). We measured their humoral responses to SARS‐CoV‐2, grouping individuals based on a BMI greater or less than 25 kg m^−2^.

**Results:**

Here, we show that an increased BMI (≥ 25 kg m^−2^), when accounting for age and sex differences, is associated with reduced antibody responses after SARS‐CoV‐2 infection. At 3 months of post‐infection, an elevated BMI was associated with reduced antibody titres. At 13 months of post‐infection, an elevated BMI was associated with reduced antibody avidity and a reduced percentage of spike‐positive B cells. In contrast, no significant association was noted between a BMI ≥ 25 kg m^−2^ and humoral immunity to SARS‐CoV‐2 at 5 months of post‐secondary vaccination.

**Conclusions:**

Taken together, these data showed that elevated BMI is associated with an impaired humoral immune response to SARS‐CoV‐2 infection. The impairment of infection‐induced immunity in individuals with a BMI ≥ 25 kg m^−2^ suggests an added impetus for vaccination rather than relying on infection‐induced immunity.

## Graphical abstract

In this study, we found decreased humoral immunity to SARS‐CoV‐2 infection in individuals with an elevated body mass index (BMI). In contrast, there was no association between increased BMI and the humoral immune response to SARS‐CoV‐2 vaccination.

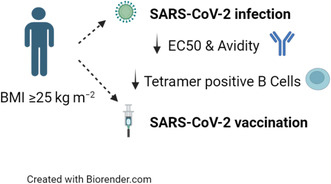

## Introduction

SARS‐CoV‐2 has resulted in > 765 million infections globally (May 2023).^1^ It is estimated that at least 10% of these cases represent re‐infection, which may occur as early as 19 days after the initial infection has resolved.^2^, ^3^ The increased rate of reinfection over the course of the pandemic has been driven by both waning immunity and the emergence of variants of concern that have evolved to evade the host immune response.^4^ Humoral immunity is essential to prevent SARS‐CoV‐2 reinfection.^5^, ^6^, ^7^ Central to an effective humoral immune response, and protection from SARS‐CoV‐2 reinfection, is the generation of memory B cells.^7^ Upon reinfection, the activation of memory B cells results in their rapid proliferation and differentiation into plasma cells that produce large amounts of high‐affinity neutralising antibodies. In addition to memory B cells, the levels of circulating antibodies provided by long‐lived plasma cells, and more specifically neutralising antibodies, present at the time of reinfection is an important correlate of susceptibility to SARS‐CoV‐2 infection and disease.^8^ This humoral response is generated from both SARS‐CoV‐2 infection and vaccination, with conflicting reports as to whether infection^9^ or vaccination^10^ elicits a stronger immune response.

There is a very little evidence to identify the host factors that are associated with the susceptibility to SARS‐CoV‐2 reinfection and the impairment of an effective humoral response. Previous studies have suggested that host co‐morbidities,^11^ and more specifically elevated BMI^12^ are associated with an increased risk of SARS‐CoV‐2 reinfection and increased disease severity upon reinfection.^13^, ^14^ This is consistent with evidence that elevated BMI impairs the humoral immune response to infection^15^, ^16^ and vaccination.^17^, ^18^ Specifically, rapid decline in humoral immune response after vaccination was observed in people with severe obesity.^18^ However, others have suggested that BMI has little effect on the humoral response to SARS‐CoV‐2 vaccination.^19^ Similarly, elevated BMI studies on the immune response to infection have typically focussed on the acute response (i.e. < 6 months post‐infection).^16^, ^20^ This raises a question on the long‐term humoral responses post infection. In individuals with BMI < 25.0 kg m^−2^, the antibody response to SARS‐CoV‐2 infection partially declines within 6 months post infection,^21^, ^22^ stabilising and remaining detectable for up to 13 months post infection.^23^ Whether antibody kinetics are altered in individuals living with elevated BMI, resulting in increased disease severity, remains to be determined. This remains even less defined in individuals who are classified as overweight but not morbidly obese (BMI 25–40 kg m^−2^). In our study, we established a cohort of people from Brisbane, Australia who had recovered from a single SARS‐CoV‐2 infection in March 2020 and were not exposed to SARS‐CoV‐2 or vaccinated in the subsequent 13 months. We also recruited individuals from Melbourne, Australia who had been vaccinated against SARS‐CoV‐2. Using these cohorts, we sought to determine whether an elevated BMI (BMI ≥ 25 kg m^−2^) affected the humoral response to SARS‐CoV‐2 infection and vaccination.

## Results

Participants who tested positive for SARS‐CoV‐2 in early 2020 were recruited for the study. To note, the SARS‐CoV‐2 infection cohort employed in the present study was relatively unique in so far as the early nature of their infection (March 2020) and the fact that vaccination did not begin in Australia until February 2021. In addition, the cohort represents humoral immune responses after a single infection, as Queensland, Australia did not have SARS‐CoV‐2 circulating in the community until state borders opened in December 2021. Blood samples from these recruited unvaccinated patients were taken from these participants at two different time points: approximately 3 months post‐infection (Visit 1) and 13 months post‐infection (Visit 2). Participants were subsequently grouped according to BMI from Visit 1 with the threshold set at 25.0 kg m^−2^, in order to assess the effects of both overweight and obesity on the humoral response, as per previous studies.^24^ We first focused on individuals who provided a blood sample at both Visit 1 and Visit 2. Participants with higher and lower BMI had equivalent sex distribution (Supplementary table 1). Importantly, these participants were recruited at similar recovery time periods from their PCR test date (Table 1). However, participants with a higher BMI were older than those with a lower BMI (Supplementary table 1).

**Figure 1:**
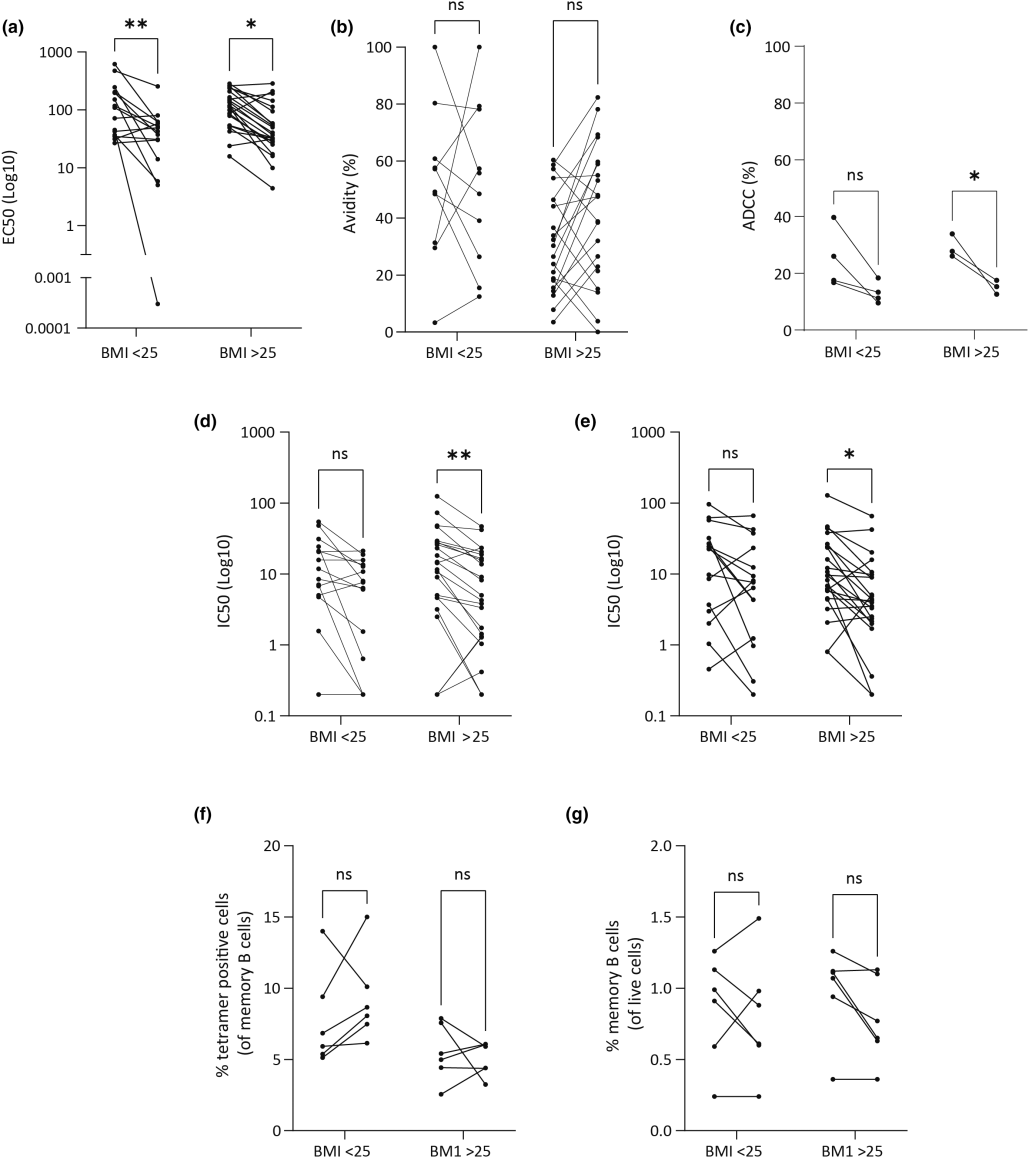
Recovered COVID‐19 patients with higher BMI experienced a higher decline in neutralising capacity. Blood samples from recovered COVID‐19 patients were collected at approximately 3 months and 13 months post SARS‐CoV‐2 infection. Serum samples were tested for IgG antibody titres **(a)** and avidity **(b)** against spike protein derived from ancestral strain. ADCC activity was also assessed with ADCC assay (Promega) **(c)**. Serum samples were also tested for neutralisation capacity against SARS‐CoV‐2 ancestral strain **(d)** and Delta strain **(e)**. PBMCs from blood samples were also collected and stained for memory B cell markers. PBMC samples were gated and analysed for spike specific cells as a **(f)** percentage of memory B cells (CD27 + CD19 + CD20+) and **(g)** memory B cells (CD27^+^ CD20^+^ CD19^+^) as a percentage of live cells. Each line represents a patient sample (*n* = 38; **a**, **b**, **d**, **e**; *n* = 8, **c**; *n* = 12, **f**, **g**). Statistical analysis was performed using a repeated measures two‐way ANOVA. *P* < 0.05 (*), *P* < 0.01 (**), *P* > 0.05 (ns).

To assess the effects of BMI on the longevity of humoral responses after infection, the total spike‐specific binding antibodies (half maximal effective concentration; EC50), neutralisation capacity (half maximal inhibitory concentration; IC50), cross‐strain neutralisation, avidity and ADCC were assessed between both visits using a paired analysis of matched samples (Figure 1). A significant decline in EC50 over time was observed in both cohorts (Figure 1a). No difference in avidity was observed (Figure 1b). However, participants with a higher BMI experienced a decline in ADCC over time whilst this was not observed in participants with a lower BMI (albeit with a reduced n number; Figure 1c). The neutralisation and cross‐strain neutralisation capacity were assessed by microneutralisation assays of ancestral and the Delta strain respectively. A significant decline in IC50 against SARS‐CoV‐2 ancestral virus (IC50 ancestral) over time was only observed in participants with higher BMI (Figure 1d). Similarly, a significant decline in IC50 against SARS‐CoV‐2 Delta (IC50 Delta) over time was only observed in participants with higher BMI (Figure 1e). To further elucidate the effects of BMI on decreased neutralisation capacity of SARS‐CoV‐2 after infection, memory B cells were assessed. No significant decline in spike specific memory B cells (Figure 1f) or total memory B cells were observed over time in either BMI high or low cohorts (Figure 1g). However, it is important to note that individuals with a higher BMI were also older than those with a lower BMI (Supplementary table 1). We thus sought to do additional analyses to account for the potential confounding effect of age on these data.

**Table 1:** COVID‐19 recovered patient characteristics at Visit 1

	BMI < 25 kg m^−2^ (*n* = 37)		BMI ≥ 25 kg m^−2^ (*n* = 45)		
	Mean (IQR)	%	Mean (IQR)	%	*P*‐value
Age	39.54 (26.25–52.27)	–	51.21 (40.33–62.41)	–	0.001
Sex (male/female)	30/7	81.08	20/25	44.44	0.0007
Time post infection (months)	2.72 (2.17–3.30)	–	2.82 (2.30–3.20)	–	0.36
BMI	22.59 (21.53–24.03)	–	29.48 (27.20–30.12)	–	< 0.0001

**Figure 2:**
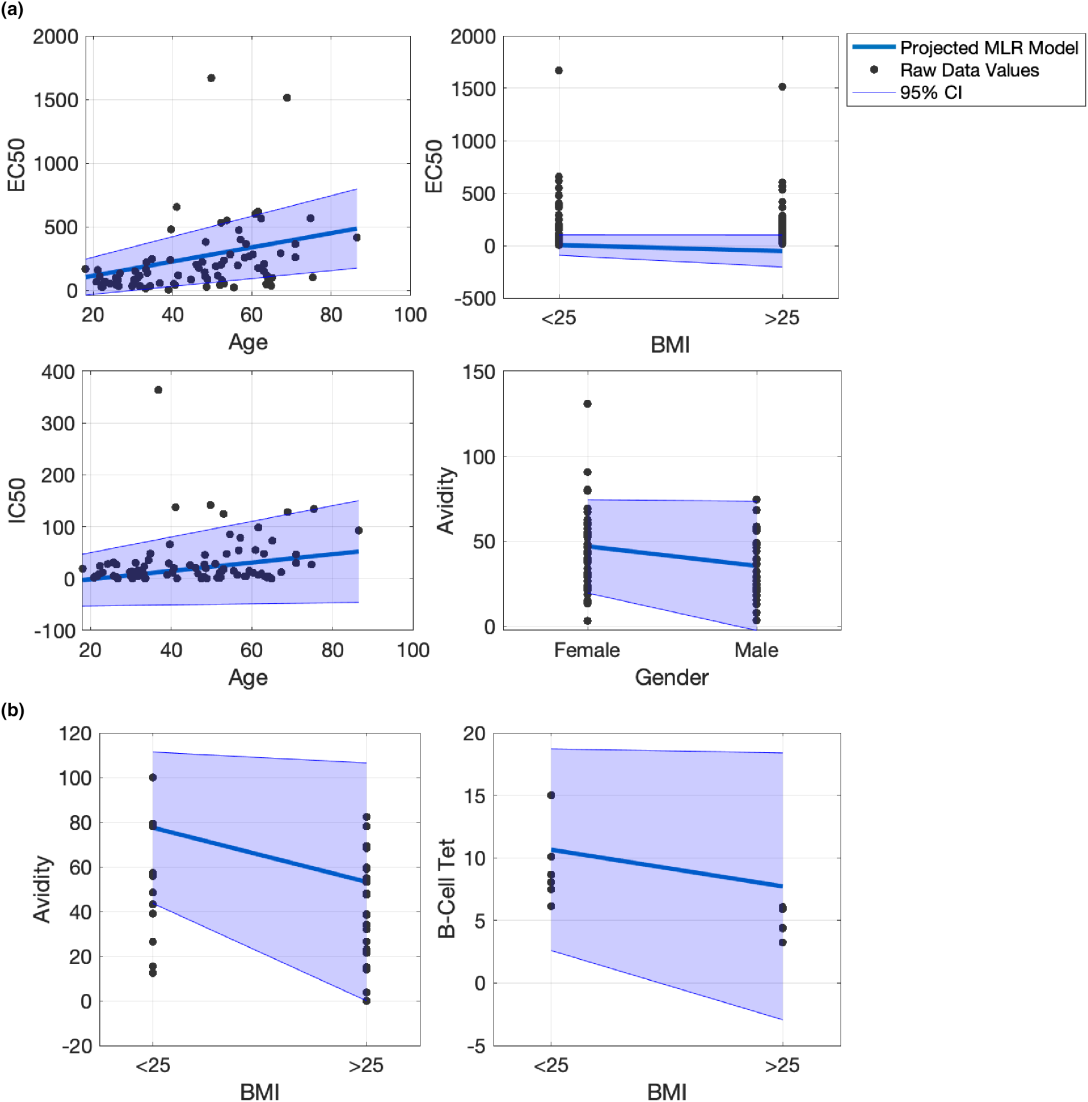
Variance weighted multiple linear regression plot of variables at Visit 1 **(a)** and Visit 2 **(b)** with coefficient values statistically significantly different from zero at 95% confidence with a standard *t*‐test.

Together with paired participant sampling at both time points, additional individual samples were collected at single time points (Tables 1 & 2). To include these additional data points in the analysis humoral responses at each visit were compared between those with a higher and lower BMI. As a result of the heteroskedastic nature of the data, and the well‐described effect of age and sex on antiviral immune responses, weighted multiple linear regression was used (Figure 2; Tables 3 & 4). At Visit 1 (Supplementary figure 2), males had a significantly lower antibody avidity than females when accounting for BMI and age (Table 3; Figure 2). Similarly, at Visit 1 older age resulted in significantly higher EC50 and IC50 (ancestral) values, when accounting for BMI and sex (Table 3; Figure 2). In contrast, elevated BMI resulted in a significantly lower EC50 at Visit 1 when accounting for age and sex (Table 3; Figure 2). At Visit 2, the only statistically significant factor associated with alterations in humoral immunity was BMI, where an elevated BMI was associated with a lower antibody avidity and a lower percentage of tetramer positive B cells (Table 4; Figure 2; Supplementary figure 3).

**Table 2:** COVID‐19 recovered patient characteristics at Visit 2

	BMI < 25 kg m^−2^ (*n* = 16)		BMI ≥ 25 kg m^−2^ (*n* = 24)		
	Mean (IQR)	%	Mean (IQR)	%	*P*‐value
Age	38.28 (23.00–56.48)	–	50.35 (43.86–60.96)	–	0.04
Sex (male/female)	5/11	70.59	13/11	52.38	0.15
Time post infection (months)	12.59 (11.90–15.20)	–	13.40 (11.50–13.88)	–	0.1
BMI	22.68 (21.71–24.10)	–	29.17 (26.96–31.57)	–	< 0.0001

**Table 3:** Comparison of humoral immunity between those with a lower (< 25 kg m^−^^2^; *n* = 45) and higher (≥ 25 kg m^−^^2^; *n* = 37) BMI at Visit 1 following SARS‐CoV‐2 infection^a^

	EC50	*P*‐value	IC50 (ancestral)	*P*‐value	IC50 (delta)	*P*‐value	Avidity	*P*‐value	ADCC	*P*‐value	% Memory B cells	*P*‐value	% tetramer positive B cells	*P*‐value
Age (Coefficient ± SE)	**5.57 (1.26)**	**2.923 × 10** ^**–5**^	**0.81 (0.36)**	**0.027**	0.43 (0.34)	0.208	−0.36 (0.17)	0.833	0.11 (0.16)	0.5	0.00 (0.01)	0.694	0.03 (0.06)	0.6
BMI (Coefficient ± SE)	**−56.85 (28.48)**	**0.049**	−5.38 (11.43)	0.639	−4.37 (12.35)	0.724	−1.80 (5.18)	0.73	7.71 (5.58)	0.188	0.26 (0.17)	0.156	−0.68 (1.60)	0.679
Sex (Coefficient ± SE)	−13.73 (23.66)	0.563	11.52 (11.19)	0.307	16.71 (15.35)	0.280	−**11.59 (5.37)**	**0.036**	−6.14 (12.39)	0.628	−0.22 (0.48)	0.648	−2.12 (4.29)	0.632

^a^ Values in bold red typeface represent statistically significant differences.

**Table 4:** Comparison of humoral immunity between those with a lower (< 25 kg m^−2^; *n* = 24) and higher (≥ 25 kg m^−2^; *n* = 16) BMI at Visit 2 following SARS‐CoV‐2 infection^a^

	EC50	*P*‐value	IC50 (ancestral)	*P*‐value	IC50 (delta)	*P*‐value	Avidity	*P*‐value	ADCC	*P*‐value	% Memory B cells	*P*‐value	% tetramer positive B cells	*P*‐value
Age (Coefficient ± SE)	0.94 (0.58)	0.114	0.14 (0.13)	0.282	−0.16 (0.18)	0.406	0.042 (0.32)	0.894	0.18 (0.11)	0.116	−0.01 (0.01)	0.096	−0.07 (0.05)	0.184
BMI (Coefficient ± SE)	0.21 (16.76)	0.989	−0.12 (3.80)	0.975	−7.04 (5.38)	0.198	**−24.30 (9.88)**	**0.019**	−1.71 (4.31)	0.699	0.07 (0.13)	0.587	**−2.93 (1.33)**	**0.049**
Sex (Coefficient ± SE)	10.42 (17.95)	0.565	−4.40 (2.27)	0.059	−8.16 (5.41)	0.139	−11.51 (11.86)	0.339	−0.24 (7.70)	0.975	0.12 (0.53)	0.828	0.82 (3.71)	0.829

^a^ Values in bold red typeface represent statistically significant differences.

Given the observed associations between an elevated BMI and an impaired humoral immune response to SARS‐CoV‐2 infection, we next sought to ascertain whether there was also an association between BMI (≥ 25.0 kg m^−2^) and humoral responses to vaccination. Forty‐seven vaccinated participants without any clinical history of positive SARS‐CoV‐2 tests, and with a limited percentage with N antibodies, were recruited for the study (Table 5). These individuals were recruited in Melbourne in 2021, at a time where there were sporadic periods of COVID‐19 outbreaks. Participants were recruited approximately 5 months after their second vaccination. Participants were grouped based on BMI threshold of 25.0 kg m^−2^. There was no significant difference in age, time post 2nd vaccination dose and vaccination type between individuals with a higher and lower BMI (Table 5). However, there was a significant difference observed in the sex distribution between both groups (Table 5). There was no difference in the proportion of people with anti‐N IgG in both groups (Table 5). In contrast to the immune responses observed following infection, no significant difference was observed in humoral immune responses to vaccination according to age, sex or BMI (Table 6; Supplementary figure 4).

**Table 5:** Patient characteristics for vaccinated samples

	BMI < 25 kg m^−2^ (*n* = 26)		BMI ≥ 25 kg m^−2^ (*n* = 21)		
	Mean (IQR)	%	Mean (IQR)	%	*P*‐value
Age	46.46 (27.25–63.75)	–	43.81 (29.00–60.00)	–	0.9282
Sex (male/female)	6/20	23.07	11/10	52.38	0.03765
Time post vaccination^a^ (months)	4.55 (3.68–5.35)	–	5.14 (3.27–5.47)	–	0.5560
N Ab detected (Y/N)	7/19	26.9	8/13	38.09	0.4140
Vaccination type^b^ (P/AZ)	15/11	57.69	14/7	66.67	0.5292
BMI	22.22 (21.00–23.95)	–	32.04 (26.8–36.7)	–	< 0.0001

^a^ Time post vaccination calculated from the second vaccination dose.

^b^ P, Pfizer‐BioNTech; AZ, Oxford/AstraZeneca (ChAdOx1‐S Recombinant vaccine).

**Table 6:** Comparison of humoral immunity between those with a lower (< 25 kg m^−2^; *n* = 24) and higher (≥ 25 kg m^−2^; *n* = 16) BMI following SARS‐CoV‐2 vaccination (approximately 5 months post second vaccination)

	EC50	*P*‐value	IC50 (ancestral)	*P*‐value	IC50 (delta)	*P*‐value	Avidity	*P*‐value	ADCC	*P*‐value	% Memory B cells	*P*‐value	% tetramer positive B cells	*P*‐value
Age (Coefficient ± SE)	−1.01 (1.12)	0.373	−0.73 (0.40)	0.072	0.22 (0.75)	0.773	−0.06 (0.27)	0.817	0.00 (0.05)	0.954	−0.03 (0.03)	0.343	0.00 (0.03)	0.888
BMI (Coefficient ± SE)	40.78 (40.26)	0.319	0.54 (11.71)	0.964	37.51 (37.29)	0.322	−17.51 (9.07)	0.065	−0.34 (0.67)	0.625	−0.88 (0.72)	0.245	−0.07 (0.56)	0.900
Sex (Coefficient ± SE)	16.23 (38.54)	0.677	2.86 (9.46	0.764	39.44 (27.67)	0.163	−9.85 (9.71)	0.320	0.50 (0.91)	0.601	−0.09 (1.10)	0.937	−0.04 (0.72)	0.957

## Discussion

Here, we provide evidence that an elevated BMI (≥ 25.0 kg m^−2^) deleteriously affects the humoral response to SARS‐CoV‐2 infection.

At Visit 1, an elevated BMI was associated with a significant reduction in EC50. At Visit 2, an elevated BMI was associated with reduced antibody avidity and a reduced percentage of SARS‐CoV‐2 spike‐specific B cells. The mechanism by which BMI deleteriously affects the humoral immune response to SARS‐CoV‐2 infection remains unclear. One possibility is that in individuals with an increased BMI, leptin overproduction from adipose tissues drives basal B cell activation *via* JAK/STAT3/5 pathway,^25^ driving overall cytokine dysregulation. Uncontrolled cytokine production could increase activation of extra follicular responses, exhausting precursors required for germinal centre processes during the formation of long‐term humoral responses (both long lived plasma cells and memory B cells) to SARS‐CoV‐2 after infection.^26^

It is important to emphasise that the SARS‐CoV‐2 infection cohort employed in the present study was relatively unique in that it represented a population of unvaccinated individuals who were infected once with SARS‐CoV‐2 and were not exposed to reinfection in the subsequent follow up period. Whilst the majority of individuals in high‐income countries now have vaccine‐induced immunity (± immunity from infection) at the time of writing, only 20.7% of people in low‐income countries have received at least one dose of a COVID‐19 vaccine.^27^, ^28^ Accordingly, concerns about the effect of BMI on infected‐induced immunity to SARS‐CoV‐2 reinfection are most relevant to people living in less economically developed countries, where vaccination rates are low and a significant number of people are living with an elevated BMI.^28^, ^29^ Whether subsequent reinfections are sufficient to overcome elevated BMI associated impairments to humoral immunity remains to be determined. Nevertheless, these findings have important implications for public policy. Namely, prior public health guidelines and now many vaccine booster recommendations, suggest assessing levels of prior SARS‐CoV‐2 immunity based on the date of prior vaccination *or* prior infection. However, these data raise the question as to whether a recent SARS‐CoV‐2 infection in individuals with an elevated BMI is an accurate indicator of robust prior immunity. These data echo other calls^18^ that individuals with elevated BMI should be presented with more personalised public health policies and vaccination schedules, in much the same way as we currently do for the elderly, those living with diabetes and the immunocompromised.

Interestingly, BMI ≥ 25.0 kg m^−2^ did not affect the humoral immune response to COVID‐19 vaccination at approximately 5 months post‐second dose of vaccination. At present, there are conflicting reports as to whether infection^9^ or vaccination^10^ elicits a stronger immune response to SARS‐CoV‐2. This question is becoming increasingly difficult to answer as fewer and fewer individuals remain with either exclusively vaccine‐induced or infection‐induced immunity. The data presented here raise the intriguing possibility that elevated BMI may have a more detrimental effect on humoral immunity to SARS‐CoV‐2 infection as opposed to vaccination. This is consistent with previous reports that the immune response to infection is different to that induced by vaccination.^30^ These data are also consistent with the findings of van der Klaauw and colleagues^18^ who recently showed that mean levels of anti‐spike and anti‐RBD IgG antibodies were similar between individuals with severe obesity (BMI > 40.0 kg m^−2^) and a healthy BMI 6 months after the second SARS‐CoV‐2 vaccine dose. However, in contrast to the findings of the present study, van der Klaauw and colleagues found that the function of these antibodies, as measured SARS‐CoV‐2 neutralisation, was reduced in individuals with severe obesity. This discrepancy may reflect the different BMI threshold of the present study (≥ 25 kg m^−2^) and that of van der Klaauw and colleagues (> 40 kg m^−2^).^18^ This is the major point of difference with our study – we simply consider the effect of a BMI that is above what is considered to be a healthy range (18.5–25 kg m^−2^). This range applies to a larger number of individuals in society and therefore, we believe that any associated immune defects have far reaching implications. With the caveat that different findings may have been observed in the present study if different timepoints post‐infection/vaccination were sampled or different BMI cut‐offs were used, our data provide an insight into the comparisons between infection‐induced and vaccine‐induced SARS‐CoV‐2 humoral responses.

In addition to BMI, age is known to affect the humoral response to SARS‐CoV‐2 vaccination.^31^ Accordingly, age was included as an independent variable in the multiple linear regression model used in the present study. Interestingly, age had no effect on vaccine‐induced humoral immunity when accounting for BMI and sex differences. This may be the result of several different features of the analysis performed herein. Firstly, whilst humoral immunity to SARS‐CoV‐2 vaccination is clearly reduced in older (i.e. > 70 years old) individuals compared to younger adults^31^ this relationship is less clear when age is treated as a continuous variable.^32^, ^33^ Specifically, whilst some studies report a negative association between age and vaccine‐induced humoral immunity^33^ others report no association.^32^ This relationship is likely to be further affected by the number of older individuals (> 70 years old) included in the analysis as well as the timepoint post‐vaccination selected for analysis. Accordingly, the effect of age on the humoral response to SARS‐CoV‐2 vaccination, when accounting for differences in sex and BMI, remains an important area for further study.

The present study has several limitations. Firstly, the number of donor samples employed herein were limited compared to other studies. This reflects the fact that there was only a limited number of individuals in Queensland, Australia who were infected with SARS‐CoV‐2 in March 2020. This population cohort was important to recruit and could not be supplemented with individuals infected later in the pandemic, as it would then not be possible to assess immunity at 13 months post‐infection in the absence of intervening reinfections or vaccinations. Indeed, several participants were lost from the Visit 2 cohort of the present study as they were amongst the earlier adopters of COVID‐19 vaccination in Australia. Accordingly, it is not possible to state whether the fewer statistically significant relationships observed at Visit 2 is indicative of a biological phenomenon or a function of the reduced sample size. Secondly, it is possible that the improved immunity associated with hybrid immunity,^34^, ^35^ or multiple reinfections can overcome the immune impairments associated with elevated BMI. Thirdly, each cohort was grouped based on their BMI during the first respective timepoint. It is possible that weight loss or gain during this study could cause unknown variations in the results. Finally, it is impossible to directly associate our findings to susceptibility to reinfection given that we did not measure T cell responses. Nevertheless, should infection‐induced immunity be impaired in individuals with a higher BMI it would suggest an added impetus for vaccination rather than relying on ‘natural immunity’. Any possible limitations of infection‐induced immunity, in particular for vulnerable population groups, remains significant for the public discourse on vaccination and is required to ensure individuals can make informed risk‐assessments of SARS‐CoV‐2 infection.

## Methods

### Ethics

Recruitment of human donors was approved by the human ethics boards of Mater Research (HREC/MML/62705), the QIMR Berghofer Medical Research Institute (Ethics: P3618), the David Serisiser Research Biobank (DSRB) (HREC/14/QPAH/275) at Mater Misericordiae Ltd and La Trobe University (Ethics: HEC21097). All methods were performed in accordance with institutional guidelines and regulations. Written consent was obtained from all study participants. To study the impact of BMI on the humoral response to SARS‐CoV‐2 infection, individuals with a history of PCR‐confirmed SARS‐CoV‐2 infection were recruited to the study from Brisbane, Australia. To study the impact of BMI on the humoral response to vaccination, individuals with a history of ChAdOx1‐S or Pfizer‐BioNTech vaccination were recruited to the study 5 months after the second dose of vaccine was administered from Melbourne, Australia. At point of recruitment, patient details including BMI were collected.

### Serum/plasma isolation from human samples

Blood samples were collected from patients recovered from PCR‐diagnosed SARS‐CoV‐2 infection at different timepoints by qualified phlebotomists. Blood was either collected in EDTA or heparin tubes (BD Biosciences, NJ, USA) or BD SST™ Vacutainers® (BD Biosciences). All blood samples were processed within 24 h of blood collection. To isolate serum and plasma, tubes containing blood samples were spun down at 2000 *g* for 10 min. Serum and plasma were heat treated at 56°C for 1 h, aliquoted accordingly and stored in −20°C prior to analysis, or in −80°C for long term storage.

### Isolation of peripheral blood mononuclear cells (PBMCs) from human blood samples

A volume of 10 mL of blood was collected in BD Vacutainer® EDTA tubes (BD Biosciences). Peripheral blood mononuclear cells (PBMC) were isolated with Lymphoprep (STEMCELL, Canada) according to manufacturer's instructions. Isolated PBMCs were subsequently frozen down in foetal calf serum (FCS; Gibco, MA, USA) containing 10% DMSO (Sigma‐Aldrich, MO, USA) at −80°C until analysis. For long‐term storage, PBMCs were stored in liquid nitrogen.

### SARS‐CoV‐2 spike HexaPro expression and purification

The design of a prefusion‐stabilised SARS‐CoV‐2 spike, called HexaPro, was performed essentially as described previously.^36^ For spike ELISAs, SARS‐CoV‐2 spike was expressed and purified as previously described.^37^ For the determination and stimulation of spike‐specific memory B‐cells, a BirA tag was added to the spike construct to enable biotinylation (required for tetramerisation). The final construct was codon optimised and synthesised (Genscript, NJ, USA) and sub‐cloned into a pHLsec vector to be expressed in HEK293T cells. HEK293T cells were grown at 37°C in a CO_2_ incubator in DMEM (Dulbecco's Modified Eagle Medium) media containing 10% foetal bovine serum (Cat: SFBS, Bovogen Biologicals) and 1% Penicillin–Streptomycin‐Glutamine (Cat: 10378016; Thermo Fisher Scientific, MA, USA). Purified DNA was incubated for 20 min with Polyethylenimine (PEI) (Cat: 408727‐100ML, Sigma‐Aldrich) at a ratio of 1:3. Following incubation, the DNA:PEI mix was added to the cell culture flasks in DMEM containing 1% FBS, 5% cocktail of supplements referred to as Mastermix (DMEM with 1% Glutamax, 1% sodium pyruvate, 1% hepes, 1% NEAA, 0.000004% beta‐mercaptoethanol), and placed back in the CO_2_ incubator for 7 days at 37°C. The supernatant was harvested and purified over affinity and size exclusion column (Cat: GE17‐5248‐02, 28 989 335; Cytiva, MA, USA). The protein was run on an SDS‐page gel to confirm purity and then biotinylated and stored at −20°C.

### SARS‐CoV‐2 spike enzyme‐linked immunosorbent assay (ELISA) and avidity assay

Anti‐spike IgG antibodies in patient samples were assessed with SARS‐CoV‐2 spike ELISAs essentially as described previously.^37^ Briefly, 2 μg mL^−1^ SARS‐CoV‐2 spike proteins were coated on ELISA plates (Thermo Fisher Scientific) overnight at 4°C. Plates were blocked and serially diluted sera or plasma samples were added to the plates and incubated at 37°C for 1 h. Spike‐specific antibodies were detected with mouse anti‐human IgG HRP (1:3000; Dako, CA, USA) for 1 h at 37°C. Plates were visualised with 2,2′‐Azino‐bis (3‐ethylbenzthiazoline‐6‐sulfonic acid; ABTS; Sigma Aldrich) in the presence of hydrogen peroxide (30% w/w; Sigma Aldrich) at 405 nm on SpectraMAX 190. EC50 values were background subtracted and calculated on Graphpad Prism 9.0.2 (Dotmatics, MA, USA) using ‘log(agonist) versus response; variable slope (4 parameters)’ function.

Avidity assays were conducted similarly to spike ELISAs described above, except samples were treated with 1 m sodium thiocyanate (Sigma Aldrich) for 15 min at room temperature before incubating with secondary antibodies. An avidity index (%) was derived from EC50 values from treated samples over EC50 values from untreated samples.

### Quantification of anti‐Nucleocapsid antibodies in sera

Anti‐Nucleocapsid IgG antibody in sera was quantified using modified ELISA as previously described.^23^ Briefly, Nunc microtitre plates (Thermo Fisher Scientific) were coated with 2000 ng of the recombinant SARS‐CoV‐2 Nucleocapsid protein per well and were incubated overnight at 4°C. Next day the plates were washed three times using Tris base with 0.1% Tween 20 (wash buffer) to remove unbound protein. After blocking for 1 h at room temperature with 5% bovine serum albumin, the plates were washed twice, and diluted vaccinated individual's sera (1:50 in 1× PBS) were added to each well in duplicate, incubated for 1 h at room temperature, and the plates were washed thrice with wash buffer. For the quantification of total anti‐Nucleocapsid IgG antibodies, 100 μL of horseradish peroxidase (HRP)‐conjugated rabbit anti‐human affinity‐purified whole IgG (1:50 000, Abcam, MA, USA) was added per well and incubated for 1 h at room temperature. The reaction was stopped using 1 m sulphuric acid and the optical density at 450 nm (OD_450_) was read using the SpectraMAX M5. The final OD_450_ values were obtained after the background subtraction of mean + 3 × standard deviation OD_450_ of four healthy individuals who were neither SARS‐CoV‐2 infected nor vaccinated.

### Cell lines

Vero E6 cells and Vero E6‐hTMPRSS2 cells were maintained at 37°C and 5% CO_2_ in Dulbecco modified Eagle medium (DMEM; Gibco) supplemented with 10% foetal calf serum (FCS; Gibco) and 1% Pen Strep Glutamine (PSG; Gibco). The cells were maintained in DMEM with 2% FCS and 1% PSG during viral infection to generate stocks.

### Viruses

SARS‐CoV‐2 isolates hCoV‐19/Australia/QLD02/2020 (QLD02; GISAID Accession ID; EPI_ISL_407896) and hCoV‐19/Australia/QLD1893C/2021 (Delta; GISAID Accession ID: EPI_ISL_2433928) were initially isolated from patient nasopharyngeal aspirates and inoculated on Vero E6 cells. Subsequently, passage 2 of these samples were kindly provided by Queensland Health Forensic and Scientific Services, Queensland Department of Health and stocks were generated in Vero E6‐hTMPRSS2 cells as described previously.^38^ Viral titres were quantified by plaque‐forming assays.^39^

### SARS‐CoV‐2 microneutralisation assays

Microneutralisation assays were performed essentially as previously described.^40^ Briefly, serially diluted sera and plasma were incubated with a multiplicity of infection (MOI) of 0.1 of virus. The mixture was transferred to Vero‐E6 cells and left to incubate at 37°C for 1 h. Following incubation, the mixture was removed and replaced with MEM (Gibco) containing 2% FCS (Gibco). The plates were left to incubate at 37°C for 1–2 days depending on the viral variant. For fixation, media was removed, and plates were fixed with 5% NBF at 4°C overnight. The fixative was removed, and cells were permeabilised with PBS containing 0.1% Triton‐X (Sigma Aldrich). Plates were washed once more in PBS and blocked in PBS containing 0.1% Tween‐20 (Sigma Aldrich) and 3% skim milk for 1 h at room temperature. The plates were subsequently incubated in PBS (Sigma Aldrich) containing 0.1% Tween‐20 and 1% skim milk with rabbit anti‐NP IgG antibody (Sino Biological, BJ, CN; 1:3000) overnight at 4°C. The plates were washed and stained with goat anti‐rabbit IgG HRP antibody (Thermo Fisher Scientific; 1:3000) at room temperature and pressure (rtp) for 1 h. Lastly, the plates were visualised with ABTS at 405 nm on SpectraMAX 190. Readings were normalised with cell and virus controls and IC_50_ readings were calculated on Graphpad Prism 9.0.2 (Dotmatics) using the ‘log(inhibitor) versus normalised response’ function.

### Antibody‐dependent cellular cytotoxicity (ADCC) assay

Antibody‐dependent cellular cytotoxicity function of recovered patient samples were analysed with ADCC reporter assay (Promega, WI, USA) accordingly to manufacturer's instructions.^41^ In summary, CHO‐K1 cells expressing Spike (Target cells) were added to serially diluted patient sera or plasma. Next, FcγR reporter bioassay effector cells (effector cells) added to the target cells and left to incubate for 6 h at 37°C. To visualise the assay, Bio‐Glo™ substrate (Promega) was added and luminescence was measured in a CLARIOstar (BMG LABTECH, UK). Non‐linear regression curve fit over a log_10_ dilution curve was performed to calculate the area under the curve using GraphPad Prism v9.

$$\;\text{Fold Inhibition}=\frac{X-“\text{background}”}{\text{average of}“\mathrm{no}\;\text{antibody}:\text{control}-“\text{background}”}$$

where X is the read for each well.

### Spike protein tetramer conjugation

The Decoy tetramer was prepared as previously described.^42^ The COVID‐19‐specific B cell tetramer was prepared as previously described,^42^ by conjugating the biotinylated HexaPro spike protein to SAV‐PE (Cat: PJRS25, Agilent [formerly ProZyme], CA, USA) at a concentration ratio of 4:1 respectively. During flow cytometric analysis, cells that bind to the HexaPro spike protein tetramer as well as the Decoy SAV‐PE*DL650 tetramer were considered Decoy‐specific and excluded from further analysis.

### B cell antibody staining and flow cytometry

Six higher BMI and six lower BMI samples were randomly chosen for B cell analysis. Staining for flow cytometry analysis was performed using cryo‐preserved PBMCs. PBMCs were stained with Fixable Viability Stain 780 (FVS780; 1:1000, BD Biosciences) diluted in PBS 2% FCS for 15 min at room temperature. Cells were washed with PBS 2% FCS and then stained with CD20‐AF700 (2H7, 1:200), CD27‐V450 (M‐T271, 1:50), CD38‐FITC (90, 1:25), IgD‐BV510 (IA6‐2, 1:50), CD10‐PE‐Cy7 (HI10a, 1:50), CD21‐FITC (B‐ly4, 1:50), FcRL5‐BV510 (509F6, 1:200; all from BD Biosciences), CD19‐BV650 (SJ25C1, 1:100), CD14‐PerCP‐Cy5.5 (HCD14, 1:100) CD16‐PerCP‐Cy5.5 (3G8, 1:100; all from BioLegend), HexaPro spike protein Tetramer‐PE (1:50) and HLA‐A2‐M1 Decoy Tetramer‐PE*DL650 (1:100; both generated in‐house) diluted in PBS 2% FCS for 20 min on ice. Cells were washed with PBS 2% FCS and fixed for 1 h using Cytofix (BD Biosciences), then washed with PBS 2% FCS and resuspended in PBS 2% FCS. Cells were analysed using an LSRFortessa X‐20 (BD Biosciences) and flow cytometry data was analysed using FlowJo™ v10.8 software (BD Biosciences). A representative gating strategy is shown in Supplementary figure 1.

### Statistical analysis

Differences between continuous variables was analysed on Graphpad v9.0.2 (Dotmatics) using a Mann–Whitney *U*‐test whilst the difference between categorical values was determined on Graphpad Prism 9.0.2 (Dotmatics) using a Chi‐squared test and paired analysis was performed with two‐way ANOVAs. Heteroscedasticity was diagnosed by inspection of the residual for a non‐weighted Multiple Linear Regression (MLR) model with Age, BMI and Gender as predictor variables for all categories of response variable (*e.g*. EC50, IC50, Avidity, ADCC and B cell percentages). For non‐weighted MLR we first fitted a non‐weighted MLR to the squared‐residuals themselves to estimate the change in variance that is occurring in the response variable. That is,

$$\left|r_{i}\right|=a_{1}\;{\mathrm{Age}}_{i}+a_{2}{\mathrm{BMI}}_{i}+a_{3}\;{\text{Gender}}_{i}$$

as a model estimate of the change in variance of our response variable resulting in input weights $w_{i}=1/r_{i}=1/\sigma_{i}^{2}$ for our weighted Multiple Linear Regression (MLR) model. The outputs from our variance‐weighted multiple linear regression model were the coefficients $b_{1}$ (Intercept), $b_{2}$ (Age), $b_{3}$ (BMI) and $b_{4}$ (Gender). We performed a *t*‐test on each coefficient in the model with null hypothesis that the coefficient is not significantly different from zero. A *P*‐value of less than 0.05 (95% confidence limit) was set to determine which variables in the variance‐weighted MLR model have a coefficient that is not significantly different from zero. All analysis was performed in MATLAB.

## Author contributions

**Marcus ZW Tong:** Data curation; investigation; methodology; writing – original draft; writing – review and editing. **Julian D. J. Sng:** Data curation; methodology. **Meagan Carney:** Formal analysis; software; visualization. **Lucy Cooper:** Formal analysis; methodology. **Samuel Brown:** Data curation; methodology. **Katie E Lineburg:** Data curation; investigation; methodology. **Keng Yih Chew:** Data curation; funding acquisition; investigation; methodology; project administration. **Neve Collins:** Data curation; methodology. **Kirsten Ignacio:** Data curation; methodology. **Megan Airey:** Data curation; methodology. **Lucy Burr:** Data curation; investigation; project administration; resources; writing – review and editing. **Briony A Joyce:** Methodology. **Dhilshan Jayasinghe:** Data curation; methodology. **Christopher LD McMillan:** Methodology; resources. **David A Muller:** Methodology; resources. **Anurag Adhikari:** Investigation; methodology; resources. **Linda A Gallo:** Investigation; supervision. **Emily S Dorey:** Data curation; investigation; project administration; resources. **Helen L Barrett:** Project administration; investigation; conceptualization; supervision; writing – review and editting. **Stephanie Gras:** Conceptualization; data curation; formal analysis; funding acquisition; investigation; methodology; project administration; resources; supervision; validation; writing – review and editing. **Corey Smith:** Conceptualization; data curation; funding acquisition; investigation; methodology; project administration; resources; supervision; writing – review and editing. **Kim Good‐Jacobson:** Conceptualization; data curation; funding acquisition; investigation; methodology; project administration; writing – review and editing. **Kirsty R Short:** Conceptualization; data curation; formal analysis; funding acquisition; investigation; methodology; project administration; resources; supervision; writing – original draft; writing – review and editing.

## Conflict of interest

KRS is a consultant for Sanofi, Roche and NovoNordisk. The opinions and data presented in this manuscript are of the authors and are independent of these relationships. Other authors declare no competing interests.

## Supporting information


Supporting information

## Data Availability

The data that supports the findings of this study are available from the corresponding author upon reasonable request.
