# Supplementary material for: Elevated BMI reduces the humoral response to SARS‐CoV‐2 infection
Source: Clin Transl Immunology. 2023 Dec 3;12(12):e1476. doi: 10.1002/cti2.1476 (PMC10693902; doi:10.1002/cti2.1476)
Supplement: Supplementary file 1 — Supporting information [file CTI2-12-e1476-s001.docx]

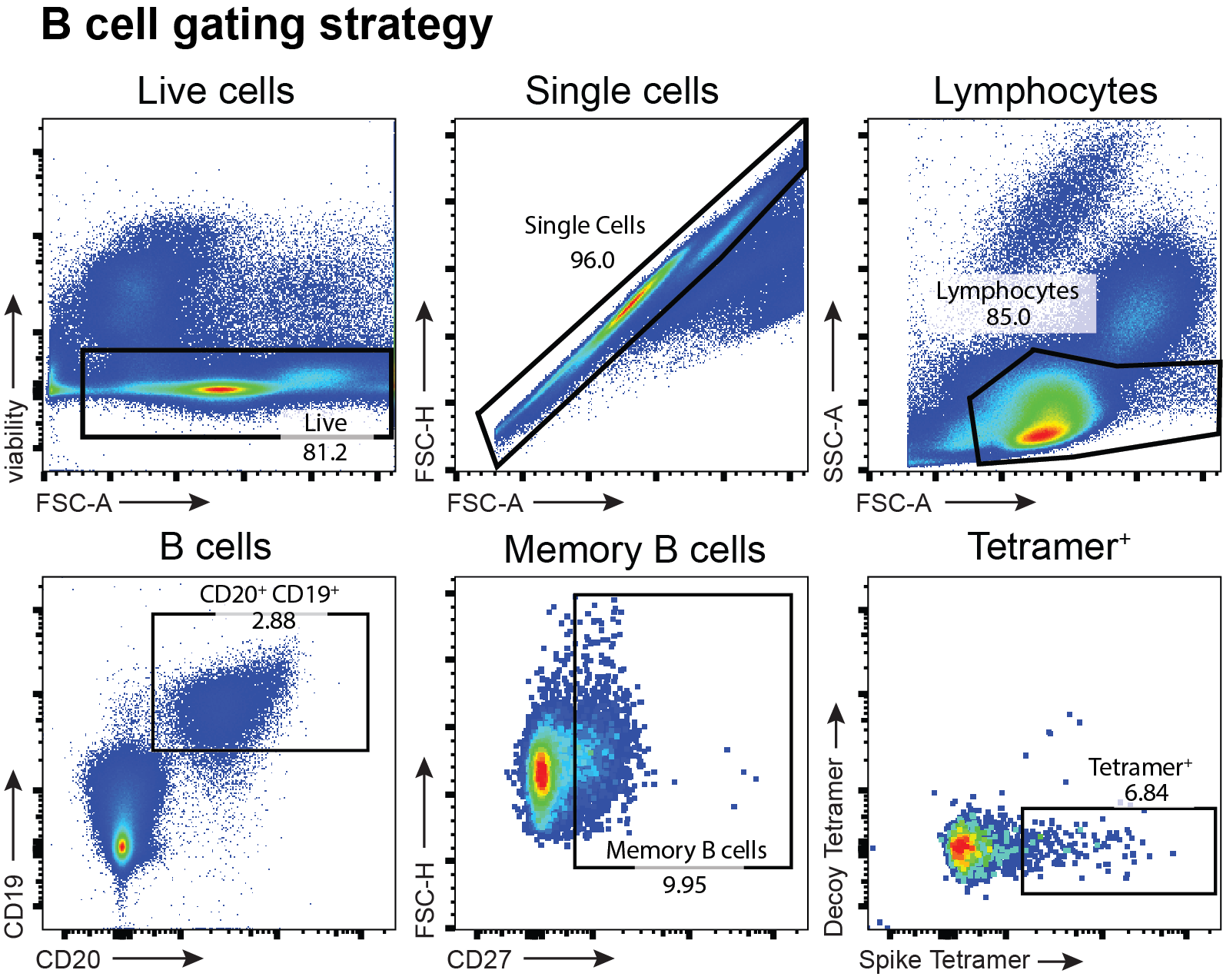


**Supplementary figure 1: Gating strategy for memory B cells and spike tetramer positive B cells.**


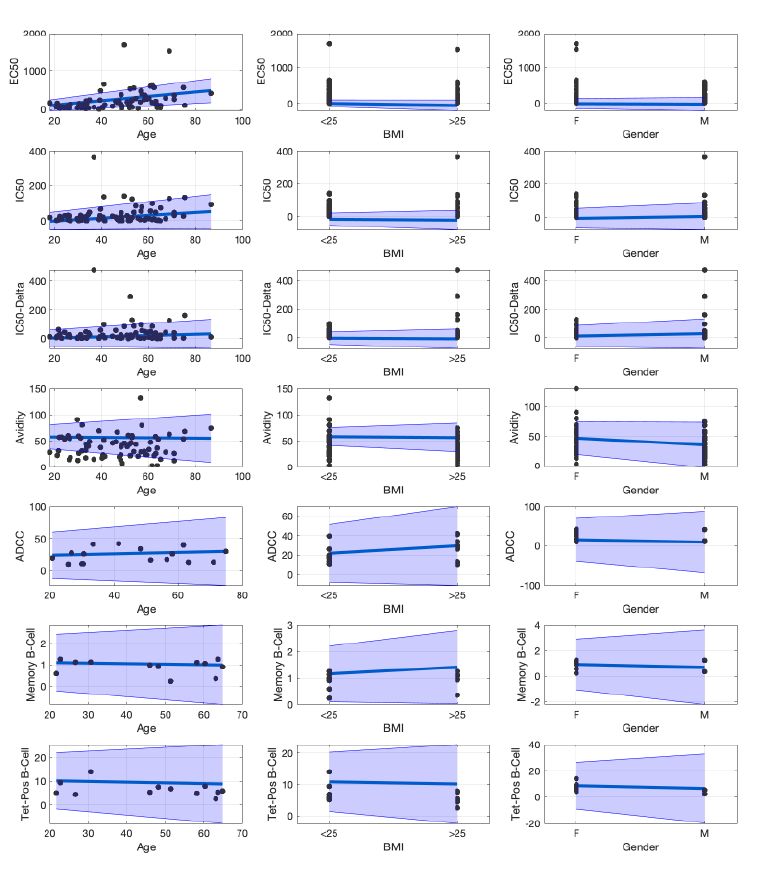


**Supplementary figure 2:** Variance weighted multiple linear regression plot of variables at Visit 1**.**


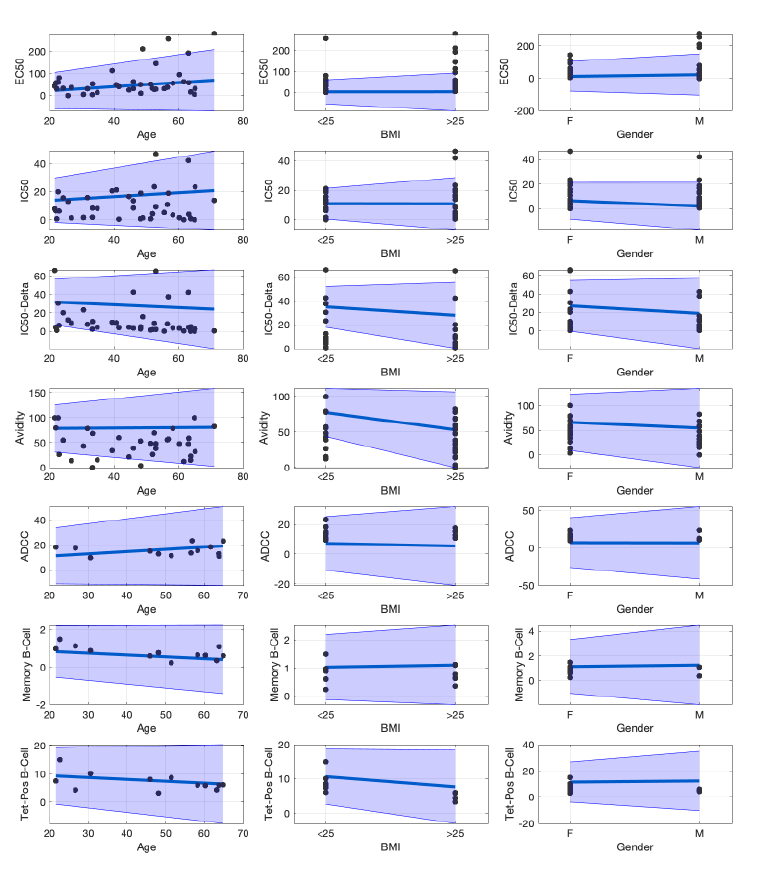


**Supplementary figure 3:** Variance weighted multiple linear regression plot of variables at Visit 2**.**


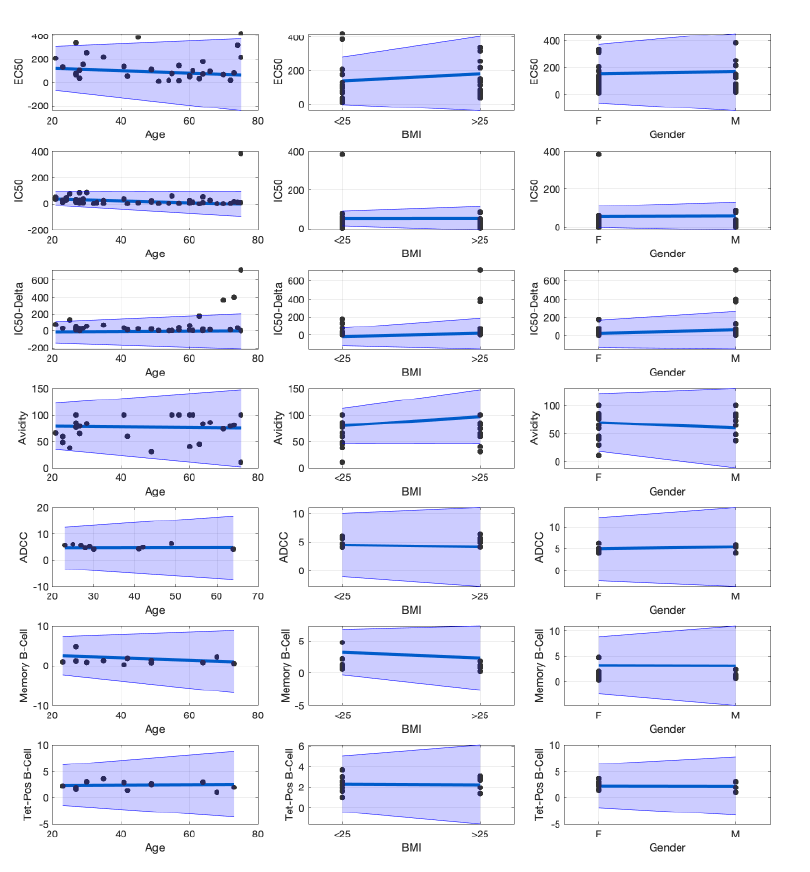


**Supplementary figure 4:** Variance weighted multiple linear regression plot of variables in vaccinated individuals**.**

**Supplementary Table 1**

|  | BMI<25 (n=15) | | BMI >25 (n=23) | | |
| --- | --- | --- | --- | --- | --- |
|  | Mean (+/- SD) | % | Mean (+/- SD) | % | p-value |
| Age | 40.20 (+/- 17.17) | - | 51.54 (+/- 12.79) | - | 0.03 |
| Sex (male/female) | 10/5 | 66.67% | 12/11 | 52.17% | 0.38 |
| Time post infection^1^(Months; Visit 1) | 2.84 (+/- 0.86) | - | 2.99 (+/- 0.92) | - | 0.46 |
| Time post infection ^1^ (Months; Visit 2) | 13.40 (+/- 1.71) | - | 12.61 (+/- 1.75) | - | 0.28 |
| BMI | 22.68 (+/- 1.38) | - | 28.88 (+/- 3.03) | - | <0.0001 |
